# Natural IgG antibodies to β amyloid are decreased in patients with Parkinson’s disease

**DOI:** 10.1186/s12979-023-00336-w

**Published:** 2023-03-11

**Authors:** Roberto Paganelli, Alessia Paganelli, Graham Pawelec, Angelo Di Iorio

**Affiliations:** 1 Department of Medicine and Sciences of Aging, University “G. D’Annunzio”, Chieti, Italy; 2 Saint Camillus International University of Health and Medical Sciences, Rome, Italy; 3 UniCamillus International Medical School, Via Di Sant’Alessandro, 8 - 00131 Rome, Italy; 4 Department of Biological, Metabolic and Neurological Sciences, University of Modena and Reggio Emilia, Modena, Italy; 5 Department of Immunology, University of Tübingen, Tübingen, Germany; 6 Health Sciences North Research Institute, Sudbury, ON Canada; 7 Department of Innovative Technologies in Medicine & Dentistry, University “G. d’Annunzio”, Chieti, Italy

**Keywords:** Parkinson's disease, Alzheimer's dementia, Anti-amyloid β, Antibodies, Neurodegeneration, Neuroimmunology

## Abstract

Natural antibodies (nAbs) against aggregation-prone proteins have been found in healthy normal subjects. These proteins likely have a pathogenetic role in neurodegenerative diseases of ageing. They include the amyloid β (Aβ) protein which may play an important role in Alzheimer’s dementia (AD), and α-synuclein, a major determinant of Parkinson’s disease (PD). We measured nAbs to Aβ in a group of Italian patients with AD, vascular dementia, non-demented PD patients and healthy elderly controls. We found that Aβ antibody levels in AD were similar to age- and sex-matched controls, but contrary to our expectations, they were significantly reduced in PD. This may identify patients that could be more prone to amyloid aggregation.

## Introduction

Parkinson’s disease (PD) is a neurodegenerative disorder characterized by the presence of protein aggregates of α-synuclein in the brain [1, 2]. The current view – similar to the amyloid theory for senile plaques formed by β amyloid (Aβ) in Alzheimer’s dementia (AD) – is that aggregation intermediates formed by these proteins are toxic to cells, whereas larger deposits of fibrils protect against neuronal damage [3–7]. It has been proposed that these amyloidogenic proteins can interact and promote each other’s aggregation [8–11]. Both Aβ and α-synuclein are targeted by natural antibodies (nAbs) present in normal healthy subjects [3, 12] as well as in patients [3, 13, 14]. In AD, levels of these nAbs were found to be lower than in healthy controls [15, 16] and most studies have confirmed this finding [17–19], despite a few discordant reports [20, 21]. The data obtained from immunization in transgenic mouse models of AD revealed that high levels of antibodies to Aβ prevented plaque formation and cognitive decline [22–25], thus leading to several but unsuccessful attempts to translate active and/or passive immunotherapy targeting Aβ to humans [12, 26, 27]. The development of monoclonal antibodies to Aβ oligomers [28] and the controversial FDA approval of aducanumab in selected cases of early mild AD [29] has sparked a debate on the role of Aβ in the pathogenesis of AD which has recently been very heated [30–33].

The significance of nAbs to α-synuclein in normal subjects and PD is much less clearly defined [13, 34, 35] and their levels have variously been found to be increased [36, 37], not differing [38] or decreased [39] in PD patients compared with controls. We set out to study the levels of nAbs anti-Aβ in the general population and patients with neurodegenerative diseases, including patients with dementia, both AD and vascular type (VaD), and a small cohort of non-demented PD cases.

## Material and methods

### Subjects

A total of 91 subjects (34 males and 57 females) was enrolled. Fifteen healthy donors aged > 65 years were selected from among volunteers at the Center of Excellence on Aging (CEA) of the UdA Foundation of the University “G. d’Annunzio” of Chieti. Other fifteen patients diagnosed with PD were selected at the Geriatric Clinic of the University Hospital. Thirty patients diagnosed with AD at the same clinic were consecutively recruited to the study. A group of 31 patients with dementia, diagnosed as vascular dementia (VaD) was also included. Diagnosis of probable AD was according to standard procedures and followed the NINCDS/ADRDA and DSM-III-R criteria [40]; Parkinson’s disease was diagnosed according to criteria valid at the time of enrollment [41] and the NINDS-AIREN criteria were applied for VaD [42], Table 1 reports the age and sex distribution of cases in each group, as well as some data of clinical importance. Age, sex, schooling and disease duration (from diagnosis) did nor significantly differ among the groups studied.

**Table 1:** Sociodemographic and clinical characteristics of patients and controls

	Healthy Controls	PD	AD	VAD	For trend *p*-value
	15	15	30	31	
Age (yy)	69.13 ± 3.55	74.25 ± 2.57	72.83 ± 1.34	75.30 ± 1.74	0.25
Male Sex (n° %)	5 (33.3)	9 (56.3)	12 (40.0)	8 (26.7)	0.31
MMSE score	26.92 ± 1.77	25.75 ± 0.65	20.19 ± 1.45	19.56 ± 0.99*	0.01
Education (yy)	3.73 ± 0.97	4.25 ± 0.30	3.53 ± 0.48	3.50 ± 0.70	0.88
CIRS score	7.07 ± 1.83	1.94 ± 0.88 *	5.67 ± 0.76	7.59 ± 0.86	0.003
Disease diagnosed (mo)	N.A	16 ± 4	30 ± 14	18 ± 11	0.77

*PD* Parkinson’s Disease, *AD* Alzheimer Dementia, *VAD* Vascular Dementia, *CIRS* Cumulative Illness Rating Scale

^*^denotes *p* < 0.05 vs control; *N.A* not applicable

All subjects underwent a geriatric examination, aimed at evaluating neurological signs and symptoms of dementia; cognitive performance was assessed by the mini mental state evaluation (MMSE) score, which was corrected for age and schooling. A radiological assessment with CT or MRI scan was also performed to assess the brain vascular disease. A significantly lower MMSE score was found in patients with VaD and AD, and cases diagnosed with PD had a better CIRS (Table 1).

The study was conducted according to the guidelines of the Declaration of Helsinki, and approved by the Ethics Committee of CEA (n. 2013/4); informed consent was obtained from all subjects involved in the study or their caregivers.

All subjects were residents in the same area of Central Italy living in the community. None was affected by major co-morbidities such as cancer, infectious or autoimmune diseases, or receiving immunosuppressive treatments at the time of recruitment. The main demographic and clinical data are reported in Table 1.

Venous blood was collected by venipuncture and allowed to clot for 30 min at room temperature, then centrifuged at 1,500 × *g* in a refrigerated centrifuge. Serum was then collected with a pipette, coded and stored in aliquots at -20 °C until tested.

### Antibody measurements

Anti-Al-42 peptide antibody was determined by ELISA on coded serum specimens from all patients and elderly controls [43]. Briefly, microtiter wells were coated with 0.1 mg/ml human Al-42 peptide (Biosource International, Camarillo, CA) in 0.1 M sodium bicarbonate buffer (pH 9.6) at 4 °C overnight. The plates were then washed three times with PBS, 0.05% Tween 20, blocked with 10% newborn calf serum (Sigma, St Louis, MO) in water for 1 h at 37 °C, and washed again three times. An affinity-purified rabbit anti-Al-42 antibody (Biosource International,Camarillo, CA) served as a positive control. The coated plates were incubated for 1 h at 37 °C with three-fold serial dilutions of serum in PBS in a 12 row of wells starting with undiluted serum. The plates were then washed and incubated for 30 min at 37 °C with alkaline phosphatase-conjugated goat anti-human IgG antibodies (ICN Biomedical, Costa Mesa, CA), washed three times, and incubated for 30 min at room temperature with p-nitrophenylphosphate (Sigma, St Louis, MO). The absorbance at 405 nm was read on an automated plate reader (Molecular Devices, Sunnyvale, CA). The titer of sera was determined in comparison to an antibody standard included on each ELISA plate.

Analysis of the differences between the groups was performed by non-parametric statistics (Fisher’s exact test and Wilcoxon’s rank sum test).

## Results

The groups did not differ for age, schooling and disease duration (from diagnosis), however, PD patients were mostly males, and with fewer comorbidities (Table 1).

**Fig. 1:**
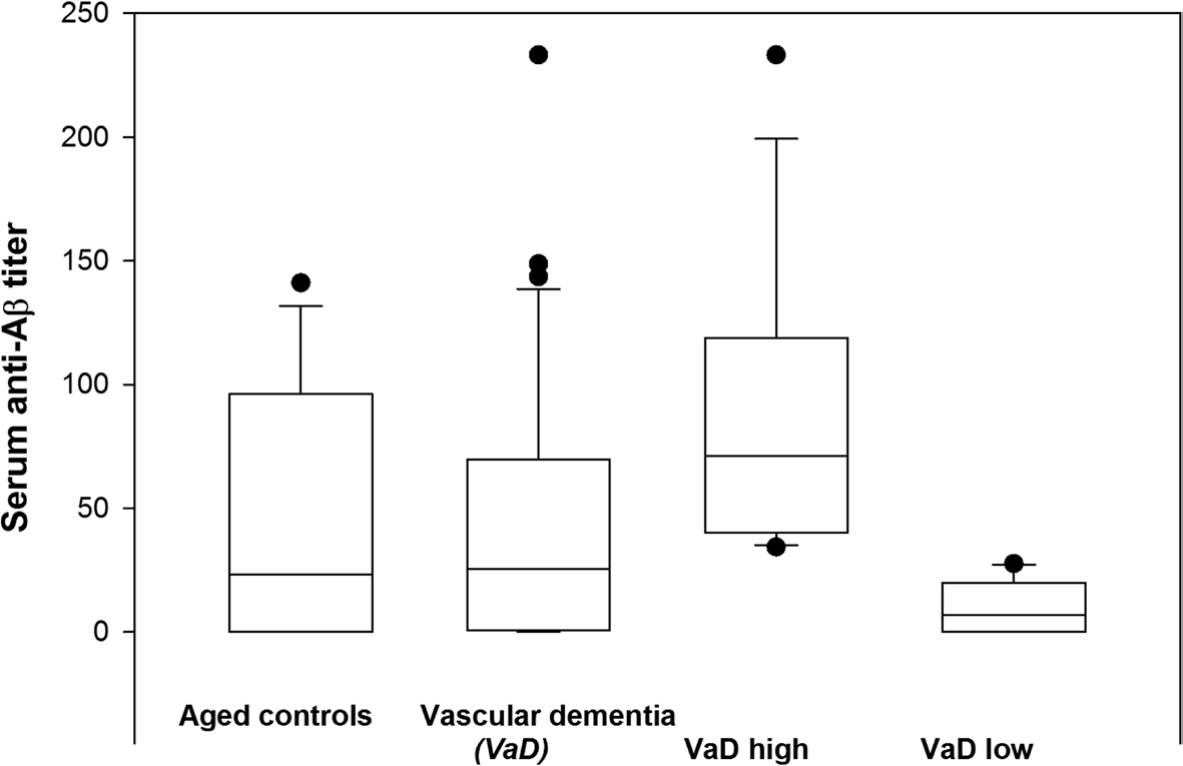
Serum levels of antibody to amyloid Aβ in 15 aged non-demented controls and 31 patients with dementia diagnosed as being of vascular origin (VaD); antibody levels did not differ between these groups. VaD cases could be subdivided in two subgroups, i.e. 14 with high (median 71.1) and 17 with low (median 6.8) antibody levels. VaD high patients had significantly more antibody to Aβ than old age controls (*p* = 0.047)

As shown in Table 2, levels of nAb to Aβ in PD patients were significantly lower (*p* < 0.05) than in healthy age- and sex-matched controls (*p* < 0.05) Their median titer was less than one third of controls (7.3 units vs. 23.2), and 73.3% of individual PD patient values were below the median of the controls. Although the median value in AD patients was 14.1 units with 63.3% of serum nAb levels below the median of the controls, this difference did not reach statistical significance. Finally, we analyzed a group of patients with VaD, which exhibited mean antibody titers similar to those found in normal controls, as expected (Fig. 1).

**Table 2:** Values of autoantibody to Aβ in healthy controls, Alzheimer’s Dementia, Vascukar Dementia and PD patients, reported as mean ± standard error (S.E.) and 95% confidence interval of the means

Groups	Number	Mean titer	S.E	95% C.I
Normal controls	15	47.846	13.433	19.041- 76.652
AD	30	40.103	12.705	14.118—66.088
PD	15	18.873*	7.744	2.264 – 35.482
VaD	31	55.932	23.543	0.391 – 154.232

^*^p < 0.05 vs other groups

However, there was some evidence for two subgroups, neatly distinguishable as clusters above and below the median. When these were examined as distinct subgroups, the titers in VaD patients with high nAb to Aβ were significantly different from healthy aged controls (*p* < 0.05). Titers measured in these Italian subjects were much lower than those reported by the same method in other studies [43]. No correlation of Aβ natural autoantibodies with age, sex and MMSE score was found.

## Discussion

In this pilot study of titers and distribution of nAbs to Aβ in the Italian population, we included both healthy aged subjects and patients with neurodegenerative diseases, both AD and PD, as well as patients diagnosed with VaD. Our hypothesis, based on published studies [5], was that anti-Aβ levels would be lower in AD compared to controls, but nAb titers were not found to be significantly different between normal subjects and AD. We had also included PD cases without clear cognitive impairment, to explore their nAbs levels to Aβ, since in this disease nAbs to α-synuclein are present [37]. Unexpectedly we found significantly reduced titers of nAbs to Aβ in these patients. There is only a small number of studies addressing this aspect, and the data are far from consistent, possibly due to technical problems in antibody detection methods [21, 43–45]. The major problem is the presence of antigen–antibody complexes in normal and patients’ sera, formed by anti-Aβ binding to soluble Aβ. This may lead to underestimation of antibody titers, and is only circumvented by acid dissociation of the bound antibody. The method has been applied to estimate the amount of anti-Aβ in commercial preparations of immunoglobulins for intravenous use (IGIV) [26, 44, 46, 47] to be used in passive immunization trials [12, 27, 48]. Aggregation of Aβ with other proteins such as tau [49] or α-synuclein [9, 11, 50] leads to possible formation of larger complexes, whereas soluble Ag-Ab complexes may trigger innate immunity and neuronal toxicity [18, 44, 51]. Recently, another protein, glycoprotein nonmetastatic melanoma protein B, has been implicated in α-synuclein induced neuronal damage [52], through colocalization detection. Another important issue is the specificity of the antibody detected in ELISA, because Aβ is known to exist in different forms with different conformational as well as linear epitopes. Protective antibodies are all those preventing the formation or promoting the dissociation of oligomeric Aβ, considered the neurotoxic form, binding to the mid-domain of the molecule [14, 21, 44, 48]. nAbs to oligomers have been studied [47, 48, 53], and they may be diagnostic tools [54–57] but we could not investigate the molecular species of Aβ targeted by our antibodies, so we can only presume that they are at least in part protective. However, the presence of high titers of antibodies to Aβ in about 45% of patients with VaD may indicate that excess antibody to some Aβ conformers has a potentially damaging role, as demonstrated for anti-Aβ in the development of the ARIA-like events characterizing cerebral amyloid angiopathy-related inflammation [24, 25, 58, 59]. These lesions have been detected also in patients treated with antibodies to Aβ [60, 61] and are probably due to rapid removal of Aβ fibrils which occurs with passive immunotherapy [28, 62]. Moreover, patients diagnosed with VaD might constitute a mixed group, some of them being both AD and VaD [63, 64], and the presence of two neatly distinct groups for anti-Aβ nAbs, as observed in Fig. 1, seems to be in agreement with such hypothesis.

Our findings show that in a phase of PD where memory and cognitive abilities are not impaired, anti-Aβ levels are greatly decreased compared to normal. This might bear on the possibility that amyloid precipitation of Aβ in fibrillar forms [65] may predate, and favour the aggregation of α-synuclein, as hypothesized in a pathogenetic scenario of PD [2, 10, 11, 49, 66–68]. Aβ aggregation is known to precipitate Tau deposition in fibrillar tangles in AD [49], and passive immunotherapy anti-Aβ might be useful in addition to anti-Tau in early phases of disease [69–71], similar to sequential anti-Aβ and anti-α-synuclein suggested in the case of PD [35, 55, 66, 72]. Passive Aβ immunotherapy has been shown to benefit with different success patients with early stage AD [30, 32, 33, 55, 73–76] since such natural antibodies may prevent oligomeric and fibrillary aggregation of neurotoxic peptides. Two recent trials of monoclonal antibodies to α-synuclein failed to meet their clinical endpoints in PD [77, 78] suggesting that targeting α-synuclein alone does not prevent progression of symptoms severity. There are possibly multiple pathways leading to neurodegeneration in PD [79], and partially interfering with one of them may not be a successful strategy [80].

We suggest that low levels of antibodies against Aβ may identify subgroups of cases in patients with PD, and possibly also AD and VaD, but their significance needs further evaluation.

## Data Availability

The data presented in this study are available on reasonable request from the corresponding author.
